# The transcriptomic signature of low aggression in honey bees resembles a response to infection

**DOI:** 10.1186/s12864-019-6417-3

**Published:** 2019-12-30

**Authors:** Clare C. Rittschof, Benjamin E. R. Rubin, Joseph H. Palmer

**Affiliations:** 1 University of Kentucky, S-225 Agricultural Science Center North, Lexington, KY 40546 USA; 2 Department of Ecology and Evolutionary Biology; Lewis-Sigler Institute for Integrative Genomics, Princeton University, Princeton, NJ 08544 USA; 3 Kentucky State University, 400 E. Main St., Frankfort, KY 40601 USA

**Keywords:** Social immunity, Colony collapse disorder, Social behavior, Nutrition, Stress, Development, Pollinator declines, Virus

## Abstract

**Background:**

Behavior reflects an organism’s health status. Many organisms display a generalized suite of behaviors that indicate infection or predict infection susceptibility. We apply this concept to honey bee aggression, a behavior that has been associated with positive health outcomes in previous studies. We sequenced the transcriptomes of the brain, fat body, and midgut of adult sibling worker bees who developed as pre-adults in relatively high versus low aggression colonies. Previous studies showed that this pre-adult experience impacts both aggressive behavior and resilience to pesticides. We performed enrichment analyses on differentially expressed genes to determine whether variation in aggression resembles the molecular response to infection. We further assessed whether the transcriptomic signature of aggression in the brain is similar to the neuromolecular response to acute predator threat, exposure to a high-aggression environment as an adult, or adult behavioral maturation.

**Results:**

Across all three tissues assessed, genes that are differentially expressed as a function of aggression significantly overlap with genes whose expression is modulated by a variety of pathogens and parasitic feeding. In the fat body, and to some degree the midgut, our data specifically support the hypothesis that low aggression resembles a diseased or parasitized state. However, we find little evidence of active infection in individuals from the low aggression group. We also find little evidence that the brain molecular signature of aggression is enriched for genes modulated by social cues that induce aggression in adults. However, we do find evidence that genes associated with adult behavioral maturation are enriched in our brain samples.

**Conclusions:**

Results support the hypothesis that low aggression resembles a molecular state of infection. This pattern is most robust in the peripheral fat body, an immune responsive tissue in the honey bee. We find no evidence of acute infection in bees from the low aggression group, suggesting the physiological state characterizing low aggression may instead predispose bees to negative health outcomes when they are exposed to additional stressors. The similarity of molecular signatures associated with the seemingly disparate traits of aggression and disease suggests that these characteristics may, in fact, be intimately tied.

## Background

Behavior often reflects an organism’s health status. For example, in vertebrates, illness and infection cause a distinct suite of behavioral responses known collectively as “sickness behavior” [53]. These phenotypes, which include lethargy, fatigue, and changes in cognitive function, are regulated by molecules that signal systemic infection to the brain [9]. Historically considered a by-product of illness, sickness behavior is now thought to be an adaptive response that helps an organism fight infection [17].

The behavioral response to illness or infection is typically generalized to multiple different infectious pathogens, possibly due to the fact that shared mechanisms communicate peripheral infection to the brain, regardless of the infectious source [17, 38]. In some organisms, even psychological or social stressors can induce sickness behavior via these same mechanisms [39]. Thus, sickness behavior reflects a cumulative physiological state that is the result of multiple different environmental stressors, acting alone or synergistically. Behavioral predictors of infection may be particularly useful in species where multiple stressors interact to varying degrees to give rise to diseased states, and therefore the source of illness may not be immediately clear and testable.

Although behavior can serve as an indicator of illness, it can also reflect disease susceptibility in healthy individuals. For example, in healthy cattle, the behavioral response to management conditions, defined as “temperament”, is correlated with the strength of the immune response to infection [14]. Stress can also result in differential activation of immune pathways in individuals with “proactive” versus “reactive” behavioral types [61]. Thus, behavioral differences among individuals can indicate variation in disease status, susceptibility, or response. In managed livestock species in particular, behavior can serve as an easily-observed and low cost first-line indicator of infection status and infection risk [23, 61, 87].

The honey bee (*Apis mellifera*) is an agriculturally managed invertebrate species showing historically high rates of colony mortality. Multiple stressors, including pathogen infection, pesticide exposure, parasite presence, and loss of floral resources due to agriculture intensification, are contributing singly and in combination to colony loss [31, 55, 80]. Recent studies suggest that, from a mechanistic perspective, these stressors behave synergistically at the colony level in part because they target similar pathways involved in immune and stress response in individual worker bees [18]. This shared physiological response to health stressors raises the possibility that a common behavioral phenotype (i.e., a sickness behavior) may be associated with disease in this species. Previous studies in the honey bee have associated some behavioral responses with specific infectious agents [37, 46, 63, 73, 90], but no generalized sickness behavior has been identified in honey bees.

Several studies have linked diverse positive health outcomes to high aggression in honey bees. These include increased colony productivity (in terms of foraging activity and brood and honey production [69, 94];), decreased *Varroa* parasitic mite loads [15, 66], and increased pesticide tolerance [66]. Honey bee aggression is exhibited by worker bees in the context of nest defense. Previous studies quantify aggression as a relative measure at the colony (using field-based assays) or individual bee (using laboratory-based assays) level [58]. Because nest defense is a collective behavior, aggression is highly socially and environmentally responsive in the honey bee [16, 36, 43, 52, 65, 66, 69, 79]. It also shows substantial variation as a function of genetic background [3, 28, 35, 42]. However, transcriptomic studies suggest that the brain molecular profile associated with high aggression shows some similarities whether the source of behavioral variation is genetic or environmental [3, 16, 67], and this brain transcriptomic state has been connected to higher physiological levels in the brain [16, 70, 71]. A shared physiological profile of high aggression, regardless of the source of behavioral variation, could explain the widespread relationships between aggression and health outcomes within and among environments and genotypes. High aggression could serve as a predictor of disease resilience (e.g., if aggression is linked pleiotropically to immune function), but low aggression may also be a response to infection (i.e., an environmentally-induced sickness behavior representing a trade-off between nest defense and immune function). In the current study, we use a molecular approach to determine whether variation in aggression resembles a generalized response to infection and parasitic feeding, recently identified in honey bees [18].

The diverse health outcomes associated with high aggression in the honey bee implicate a number of tissues including the brain as a regulator of behavior, the fat body, a metabolic tissue that is involved in immune response [88], and the midgut, which is involved in pesticide detoxification [54]. Communication between peripheral, immune responsive tissues and the brain is characteristic of sickness behavior in vertebrates [17], but in the context of honey bee aggression, no study has evaluated tissues other than the brain to establish a role for peripheral systems in behavioral variation.

Here we sequence RNA extracted from the brain, fat body, and midgut of worker bee siblings that differ in aggression as a result of their developmental experience [66]. In a previous study, we fostered these siblings in high and low aggression colonies during their egg, larval, and pupal stages. We removed these bees from the colonies the day prior to adult emergence, and allowed bees to emerge in a laboratory incubator in order to isolate the impacts of developmental environment on adult behavior. Once these bees were 8-day-old adults, we either assayed them for aggression in small groups, or preserved them for molecular analysis. We showed that siblings that developed in high-aggression colonies were more aggressive and more pesticide tolerant as adults compared to ones that developed in low-aggression colonies. Here we report the results of an RNAseq analysis of individual bees preserved from these same treatments.

In our analysis, we first assess evidence of differential viral or bacterial infection in our samples, based on RNA abundance. We then determine whether genes differentially expressed as a function of aggression are significantly enriched for transcripts identified in a recent meta-analysis to be consistently differentially regulated by pathogen infection and parasitic feeding [18]. We further assess overlapping genes for directional concordance based on the hypothesis that low aggression resembles an infected state, i.e., that genes upregulated with infection are upregulated in low aggression bees, and that genes downregulated with infection are downregulated in low aggression bees.

We take a similar approach to evaluate the relationship between brain gene expression and aggression as a function of the developmental environment. We assess whether differentially expressed genes in our study are enriched for those rapidly modulated by social alarm cues indicating a predator threat, genes modulated by prolonged exposure to aggressive nestmates during adulthood, or genes modulated in the context of behavioral maturation, the process by which adult honey bees progress through different behavioral tasks as they age (older adult bees are generally more responsive to aggressive cues [6]). These comparisons allow us to assess how the molecular state associated with developmentally-induced variation in aggression is similar to and distinct from other contexts for environmentally-induced changes in behavior. Such comparisons are relevant to understanding more broadly how aggression, a highly dynamic, socially-regulated behavioral phenotype that reflects the defensive needs of the colony, is related to disease.

Although our study is correlative, it is a critical step towards explaining the relationship between aggression and health resilience. Specifically, we are using changes in gene expression to determine how a behavioral phenotype like aggression predicts susceptibility to health stressors. By assessing evidence for pathogen infection, we can also determine whether low aggression is a sickness behavior, perhaps representing a trade-off between aggression and immune system activity.

## Results

### Differential expression analysis

We performed an analysis to determine which genes were differentially expressed among siblings who developed in a high versus low aggression environment. We previously showed that bees collected at the same time as these molecular samples showed variation in aggression that matched their developmental environment. We analyzed differential gene expression on a per-tissue basis. 85, 1571, and 312 genes were differentially expressed in the brain, fat body, and midgut tissues, respectively (Additional file 1: Tables S1, S2 and S3). Genes in the brain were significantly biased towards upregulation in low aggression bees (81%, binomial test, *P* < 0.0001), while direction of expression was not significantly biased in the fat body (49% upregulated, binomial test, *P* = 0.27) or midgut (55%, binomial test, *P* = 0.07).

To describe the function of genes related to aggression, we performed a Gene Ontology (GO) analysis followed by a REViGO analysis of significant GO terms (Benjamini-Hochberg corrected *P* < 0.05). REViGO clusters GO terms on the basis of semantic similarity to identify major patterns in long GO term lists [81]. Differentially expressed genes in the brain were significantly enriched for 23 GO terms (Additional file 1: Table S4). The REViGO clustering analysis showed clusters of processes and functions related to chaeta morphogenesis, disaccharide transport, and RNA polymerase II regulatory region sequence-specific DNA binding. These results suggest strong roles for transcriptional regulation, sensory development, and carbohydrate metabolism in differentiating brain gene expression profiles for high versus low aggression bees. Differentially expressed fat body genes were significantly enriched for 188 terms (Additional file 1: Table S5), including processes and functions associated with nucleotide and energy metabolism, and transporter activity. Only one GO category, toxin activity, was significantly enriched among differentially expressed midgut genes.

All pairwise tissue comparisons showed some overlap in genes differentially expressed as a function of aggression, with the strongest similarities between the midgut and fat body. Eight genes were differentially expressed in both the fat body and brain (enrichment test for significant overlap, *P* = 0.79), and seven of eight genes showed the same direction of change as a function of aggression (binomial test, *P* = 0.07). For the brain and midgut, six genes overlapped (*P* = 0.006) with five of six genes showing the same direction of change (binomial test, *P* = 0.22). Seventy-six genes overlapped between the fat body and midgut (hypergeometric test, *P* < 0.0001), with 71 showing the same direction of regulation across these two tissues (binomial test, *P* < 0.0001). This suggests robust expression similarity across these tissues. Only a single gene, a homeobox transcription factor (GB51409) was differentially expressed across all three tissues.

### Relationship between low aggression and disease state

#### Are low aggression bees infected with a pathogen?

We detected five bacterial pathogens, four fungal pathogens, deformed wing virus, and acute bee paralysis virus in all three tissues in at least one individual in our study (Table 1). No pathogen was detected in every individual, but most pathogens were present in at least one tissue in every individual. No pathogen was significantly more abundant or more likely to be present in low aggression samples (Additional file 1: Table S6, S7 and S8), suggesting molecular differences as a function of aggression were not caused by acute pathogen infection.

**Table 1:** The median number of reads (per million in the library) that mapped to each pathogen in high and low aggression samples. Pathogen presence and abundance was assessed from RNAseq reads that failed to map to the honey bee genome. Numbers listed after tissue types show the sample sizes for high and low aggression individuals sequenced

		Median reads mapped per million (high/low aggression)		
Pathogen	Type	Brain (13/12)	Fat body (11/11)	Midgut (13/12)
*Melissococcus plutonius*	Bacteria	1.41/1.23	1.76/1.26	2.14/2.67
*Paenibacillus larvae*	Bacteria	1.00/0.76	0.78/1.23	1.39/2.06
*Serratia marcescens*	Bacteria	3.34/2.62	6.53/4.62	9.07/5.28
*Spiroplasma apis*	Bacteria	0.61/0.52	0.46/0.67	0.81/0.90
*Spiroplasma melliferum*	Bacteria	3.55/3.30	1.54/2.00	1.36/1.55
*Ascosphaera apis*	Fungus	1008.72/981.31	734.12/731.58	595.61/647.32
*Aspergillus flavus*	Fungus	2428.87/2208.51	1918.50/1893.73	2986.38/2174.00
*Aspergillus fumigatus*	Fungus	1217.69/1116.03	868.29/926.83	1584.81/1117.31
*Aspergillus niger*	Fungus	2436.75/2261.06	1754.62/1822.11	3414.74/2413.54
Acute bee paralysis virus	Virus	0/0	0/0	0/0
*A. mellifera* filamentous virus	Virus	13.79/20.78	0.67/0.93	1.69/1.48
Black queen cell virus	Virus	0/0	0.12/0	0.07/0
Chronic bee paralysis virus	Virus	0/0	0/0	0/0
Deformed wing virus	Virus	0.03/0.03	0.25/0.80	0.03/0.14
Israel acute paralysis virus	Virus	0/0	0/0	0/0
Kashmir bee virus	Virus	0/0	0/0	0/0
Sacbrood virus	Virus	0/0	0/0	0/0
Slow bee paralysis virus	Virus	0/0	0/0	0/0

#### Does aggression correspond to variation in immune activity?

To evaluate whether the molecular patterns associated with low aggression resemble a diseased state, we compared our differentially expressed gene lists with a recently published meta-analysis that identified genes for which expression changed in response to pathogen infection or parasitic feeding across a variety of tissue types and combinations, including the whole bee, whole abdomen, fat body, midgut, and brain [18]. This meta-analysis identified 57 genes consistently upregulated and 110 genes consistently downregulated in response to infection, whether the source was parasitic mite feeding, viral or fungal infection, or some combination. We performed two enrichment tests per tissue type in our study, evaluating significance in overlap between our differentially expressed gene lists and the up and downregulated genes from Doublet et al. [18]. We also evaluated directional concordance, with the hypothesis that genes upregulated with infection would be upregulated in low aggression bees, and genes downregulated with infection would be downregulated in low aggression bees if it is a phenotype associated with disease.

In the brain, only one differentially expressed gene overlapped with the Doublet et al. [18] upregulated gene list, significant overlap due to the relatively small number of differentially expressed genes in this tissue (particularly after list conversion, see METHODS, hypergeometric test, *P* = 0.03). This single gene, GB42523 (an uncharacterized non-coding RNA), was upregulated in low aggression bees, consistent with the hypothesis that low aggression resembles a diseased state. Two genes overlapped with the downregulated Doublet et al. list (*P* = 0.01). GB45913 (lethal (2) essential for life, related to heat-shock proteins) was downregulated in low aggression bees, while the second, GB50116 (chymotrypsin inhibitor) was upregulated in low aggression bees.

In the fat body, 13 genes overlapped with the 56 upregulated genes in the Doublet et al. list (Table 2). This overlap was statistically significant (hypergeometric test, *P* = 0.04). Moreover, 10 of the 13 genes were upregulated in low aggression bees, 77% directional concordance with the hypothesis that the fat body molecular signature of low aggression resembles a diseased state (a significant directional bias, binomial test, *P* < 0.05). Seventeen genes overlapped with the downregulated Doublet et al. list (out of 110), but this was not statistically significant (*P* = 0.39), nor was the degree of directional concordance (Table 3, 64%, *P* = 0.17). Notably, one gene, hymenoptaecin, was listed on both the up and downregulated gene lists in Doublet et al. [18].

**Table 2:** Genes differentially expressed in the fat body as a function of aggression and upregulated as a result of immune activation [18]. The degree of overlap with the 57 Doublet et al. genes is significant (*P* = 0.01). Ten of thirteen genes show directional concordance (77%, one-tailed binomial test, *P* < 0.05)

BeeBase ID	Gene name	Up in Low	RefSeq ID
GB54571	FACT complex subunit Ssrp1	N	726058
GB40390	Mitochondrial sodium/hydrogen exchanger 9B2-like	Y	725900
GB41361	Cytochrome b5-like	Y	724654
GB51223	Hymenoptaecin	Y	406142
GB41428	Def-1	Y	406143
GB44824	Corazonin receptor	Y	409042
GB48134	Lactate dehydrogenase-like	Y	411188
GB47618	Def-2	Y	413397
GB51482	Unchar LOC413858	Y	413858
GB54097	Malvolio	Y	494509
GB49709	Coiled-coil domain-containing protein 86	N	551400
GB53565	Endochitinase	N	551600
GB40148	Cytochrome b561 domain-containing protein 2-like	Y	100576555

**Table 3:** Genes differentially expressed in the fat body as a function of aggression and downregulated as a result of immune activation [18]. The degree of overlap with the 110 Doublet et al. genes is not significant (*P* = 0.39), nor is the direction of concordance (*P* = 0.17)

BeeBase ID	Gene name	Up in Low	RefSeq ID
GB49544	Vitellogenin	N	406088
GB51223	Hymenoptaecin	Y	406142
GB52023	Cytochrome P450 6AQ1	N	408383
GB43006	Glucose dehydrogenase [FAD, quinone]	N	408603
GB50423	Uncharacterized LOC408807	Y	408807
GB40976	Heat shock protein 90	Y	408928
GB49504	Alpha-tocopherol transfer protein-like	Y	409740
GB50218	Ornithine aminotransferase, mitochondrial	N	410583
GB45499	Sodium-coupled monocarboxylate transporter 2	N	410683
GB40227	Facilitated trehalose transporter Tret1	N	412797
GB46223	Odorant binding protein 14	N	677673
GB49331	Leucine-rich repeat neuronal protein 1	N	724772
GB43823	Chemosensory protein 1	Y	725382
GB40212	Protein mesh	N	725498
GB47974	Carboxylesterase	N	726134
GB42797	Circadian clock-controlled protein	N	726981
GB43515	Pancreatic lipase-related protein 3-like	Y	727032

In the midgut, 3 genes overlapped with the 56 upregulated Doublet et al. [18] genes (hypergeometric test, *P* = 0.06). These were GB42523 (uncharacterized), GB48134 (L-lactate dehydrogenase), and GB44112 (melittin); all three were upregulated in low aggression bees. Seven genes overlapped with the downregulated Doublet et al. [18] genes (hypergeometric test, *P* = 0.007). These were GB59710 (protein scarlet), GB42053 (NPC intracellular cholesterol transporter 2), GB47279 (cytochrome P450 6 k1), GB40976 (HSP90), GB52023 (cytochrome P450 6AQ1), GB49854 (alpha-amylase), GB44549 (glucose oxidase). Five of seven showed concordance with the hypothesis that low aggression resembles a diseased state (a non-significant result, *P* = 0.23). Overall, across all three tissues, we find evidence to support the hypothesis that the molecular signature of low aggression resembles the molecular signature of pathogen infection and parasitic feeding.

#### Does the molecular signature of aggression include predator-responsive genes?

The pre-adult developmental environment could cause low aggression by modulating the baseline expression of genes that are responsive to alarm cues. To test this possibility, we compared our list of genes differentially expressed in the brain as a function of aggression to genes differentially expressed following alarm pheromone exposure [3], which induces a rapid, aggressive anti-predator response. Two genes (GB40074, hormone-like receptor in 38 and GB45913, protein lethal(2) essential for life) overlapped, a non-significant result (*P* = 0.09).

#### Do pre-adult and adult colony environment effects on aggression share a molecular signature?

Using a series of experiments that involved housing adult worker bees from high and low aggression strains in colonies with the opposite genotype and aggression levels, Alaux et al. [3] found that certain genes in the brain are differentially expressed as a consequence of colony environment, irrespective of individual genotype. This social treatment also affected expression of aggression [3, 43]. We compared genes differentially expressed as a function of adult colony environment to those differentially expressed as a function of aggression in our study to determine if similar genes are regulated by the adult and pre-adult social environment. Four genes were shared across these lists (GB54316, cardioacceleratory peptide receptor, GB43805, membrane metallo-endopeptidase-like 1, GB41643, blue sensitive opsin, GB54675, uncharacterized), but this degree of overlap was not significant (*P* = 0.19).

#### Does variation in aggression share a molecular signature with adult behavioral maturation?

Adult workers shift tasks as they age, a process called behavioral maturation. This process is influenced by social and environmental cues [41, 75], genotype [28], and various stressors [29, 93]. Older workers performing foraging tasks are typically more aggressive than younger hive bees, and accelerated transition to foraging is associated with higher aggression [28]. Juvenile hormone regulates both behavioral maturation and larval development, suggesting these processes, and their relationship to aggression, could be mechanistically linked. To assess whether the molecular signature of aggression in our study resembles the signature of adult behavioral maturation, we compared differentially expressed genes in the brain to those differentially expressed between foragers (older adult workers) and nurses (younger adult workers) [3]. We found that seven genes (Table 4) overlapped between these lists, a statistically significant result (*P* = 0.01). Five out of seven genes showed directional concordance between low aggression bees and younger nurse bees, suggesting low aggression bees may be developmentally delayed. However directional concordance in this case was not statistically significant (*P* = 0.23).

**Table 4:** Genes differentially expressed in the brain as a function of aggression and differentially regulated in the brain between older, foraging adults compared to younger nurse bees. The degree of overlap between these two gene sets is significant (*P* = 0.01), but there is no significant directional bias (*P* = 0.23)

BeeBase ID	Gene name	Up in nurse	Up in Low	RefSeq ID
GB55170	Uncharacterized	N	Y	724335
GB43848	Glucose-induced degradation protein 8 homolog	N	N	409454
GB40074	Hormone-like receptor in 38	N	N	551592
GB55757	Uncharacterized	Y	Y	100577047
GB52702	Facilitated trehalose transporter Tret1	N	Y	552592
GB45913	Protein lethal(2) essential for life	N	N	724488
GB51551	Myophilin	N	N	408572

## Discussion

**Fig. 1:**
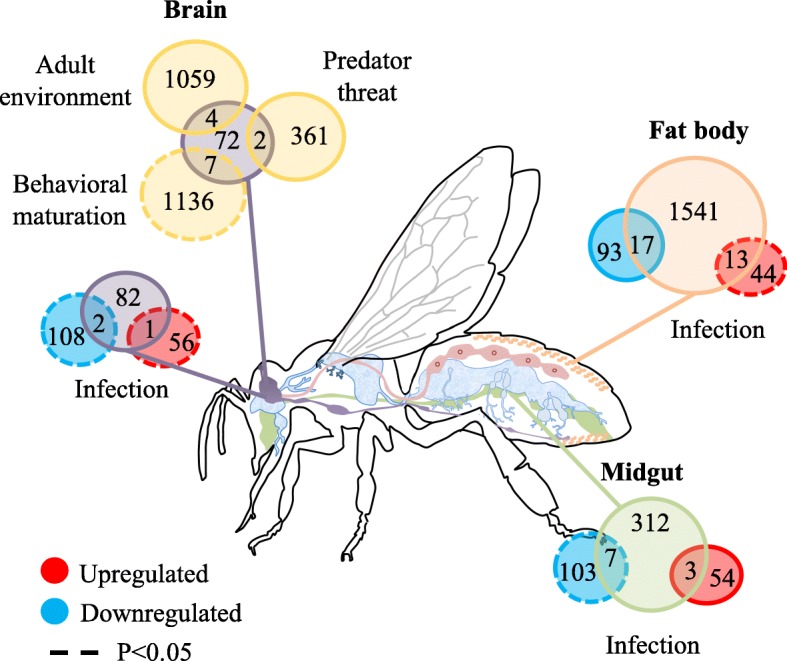
This schematic provides a summary of enrichment analysis results in the present study. "Infection" (Brain, Fat body, Midgut) indicates the tissue-specific comparison of genes differentially expressed as a function of aggression in the current study to genes differentially expressed as a function of infection in [18]. "Adult environment", "Predator threat", and "Behavioral maturation" indicate brain enrichment comparisons of genes differentially expressed as a funciton of aggression in the current study with a previous microarray study [3], which evaluated genes differentially expressed following exposure to aggression-inducing alarm cues (Predator threat), exposure to a high versus low aggression environment as an adult (Adult environment), and adult behavioral changes with aging (Behavioral maturation). In our data analysis, gene lists up and downregulated with infection or parasitic feeding were analyzed separately, while other aggression comparisons in the brain were analyzed irrespective of expression direction because the brain differentially expressed gene list in our study was short. Significant enrichment is indicated by a dotted circle. Gene numbers listed for each tissue sum to the total differentially expressed genes in the current study, not the total genes incorporated in the enrichment analyses; gene conversions across studies, spanning multiple genome versions, gene sets, and gene expression analysis methods, decreased the universe of genes used for enrichment analyses

Our results show that environmentally-induced variation in aggression in honey bees is correlated with a molecular phenotype that resembles the signature of pathogen infection and parasitic feeding (Fig. 1). We found significant enrichment for infection-responsive genes in all three tissues, and in the fat body, and to some degree the midgut, we find evidence of directional concordance consistent with the hypothesis that low aggression resembles a diseased or parasitized state. However, we found little evidence of acute infection in low aggression individuals; the abundance of infectious agents, as measured by the presence of pathogen-derived sequence reads, was not higher in these bees. We also found limited evidence that the brain molecular signature in the current study is enriched for genes modulated by social cues that induce aggression in adults. Interestingly, we do see a signature of carbohydrate metabolism among genes differentially expressed in the brain in our study, consistent with studies linking glycolysis and oxidative phosphorylation to social and environmental modulation of aggression [16, 52, 65, 70, 71]. Finally, enrichment analyses provide some support for the hypothesis that variation in aggression in our study reflects variation in the pacing of behavioral maturation in adults. Our study provides evidence that the molecular state associated with low aggression resembles a diseased state, providing a potential physiological link between high aggression and resilience to health stressors.

Although our method for assessing pathogen infection is indirect and limited to a transcriptional signature in specific tissues, at least some bacterial, fungal, and viral pathogens were found in every individual examined, suggesting that these data can be used to estimate infection load. Using these estimates, we find no significant differences in the abundance of any pathogen between high and low aggression bees, indicating that variation in aggression as a result of developmental environment is not the result of differences in infection rates. The set of pathogens we considered includes those that are known to commonly infect honey bees [13, 20, 25], including Deformed Wing Virus, a strain of which has been associated with aggression in a previous study ([24]; see also [72]). This approach for estimating infection rates may be useful for studies of honey bee behavior moving forward; despite the use of polyA-enrichment for extracting mRNA, substantial numbers of both bacterial and viral reads were present in our RNAseq datasets.

It is important to note that our current study focused on environmentally-induced variation in behavioral and molecular phenotypes, specifically the impacts of developmental social environment on aggression and gene expression. Additional studies are needed to determine how genetically-based variation in aggression corresponds to the disease-related phenotypes we evaluate here. Other studies have noted parallels in the molecular signatures of aggression arising from genetic and environmental factors [3, 27], and genetic variation in aggression is associated with variation in certain health-related phenotypes. For example, highly aggressive genotypes are known to express *Varroa* mite-resistant traits at higher levels compared to more docile strains [15]. Few studies have evaluated the relationship between aggression and health phenotypes while considering the underlying causes of behavioral variation (i.e., genotype, environment, or their interaction), an important topic for future work.

Assuming infection-related gene expression patterns reflect immune system activity, one interpretation of our data is that the relationship between aggression and infection-induced gene expression represents an investment trade-off between two energetically demanding phenotypes: low aggression bees are investing more in immune function at the expense of aggression [1]. Rather than a reflection of infection status at the time we collected these bees (as we find no evidence of infection in low aggression bees), this differential investment could be protective against future infections. In the current study, we did not directly evaluate resilience to health stressors as a function of aggression, and so it is possible that low aggression bees here are protected against infection. However, in our previous study, low aggression bees were more susceptible to topical pesticide treatments, and low aggression hives generally had higher parasitic mite levels [66]. Other studies show that at the colony level, low aggression hives have worse survival outcomes and lower foraging activity [69, 94]. Together these results suggest low aggression bees are stress susceptible, and not resilient, which contradicts the hypothesis that low aggression represents a trade-off of behavioral expression for immune function.

We cannot exclude the possibility that low aggression bees are perceiving and responding to pathogen risk and activating their immune system to successfully counteract and eliminate infection. It is also possible that immune system dynamics in relationship to aggression vary with the type of stressor experienced [21, 33]; low aggression bees may be more susceptible to a pesticide, but less susceptible to a pathogen (the latter was not measured). Furthermore, our approach does not explicitly examine the extent of the change in expression of each gene associated with infection and aggression variation. An enrichment approach for differentially expressed genes only accounts for the degree of gene expression difference in as much as it impacts the significance of the treatment effect. We expect that the degree of change in gene expression associated with aggression is more limited than for infection; it could be that this expression variation is below a threshold that is biologically relevant to immune system function. On-going studies are examining how baseline variation in aggression predicts immune gene expression and the dynamic response of gene expression to pathogen infection (Rittschof et al., unpublished).

In the brain, we found evidence that genes differentially expressed between high and low aggression siblings are significantly enriched for genes differentially expressed between nurse and forager worker bees [3, 89]. Worker bees change tasks as they age, a process known as behavioral maturation. Young workers perform tasks inside the hive including nursing, while older bees perform tasks outside of the hive including energetically-demanding foraging and defensive behaviors [91]. Thus, our results suggest that the pre-adult developmental environment, and resulting variation in aggression and pesticide tolerance, could be related to variation in adult developmental pacing. Older bees are typically more aggressive, and in keeping with this, a majority of overlapping genes support the hypothesis that high aggression bees show accelerated behavioral maturation, although this directional bias was not significant.

Behavioral maturation is impacted by social factors in healthy individuals [50], but certain stressors, including food limitation, disease infection, or social isolation accelerate behavioral maturation [29, 40, 75, 83, 84, 93]. There are some exceptions to this pattern, i.e., cases in which stress delays behavioral maturation [69]. Accelerated behavioral maturation has also been associated with stress resilience. For example, Wang et al. [86] showed that nutritional stress during the larval stage caused same-aged adult bees to show both increased titers of juvenile hormone and starvation resistance. Because juvenile hormone titers increase as adult worker bees age [40], larval nutritional stress appears to both accelerate behavioral maturation and confer stress resistance. The current study is one of the few that has examined how the pre-adult environment, including maternal or larval stress, impacts adult behavior, physiology, and gene expression in honey bees [56, 60, 66, 76]. It is possible that stressors experienced at the pre-adult stage have effects distinct from those experienced during adulthood.

Aggression is modulated by the social environment experienced throughout adulthood, but we found little overlap with the molecular signature of this effect in our study. In adults, genes rapidly modulated by alarm pheromone, an aggression-inducing social cue, and genes modulated by long-term residence in a highly aggressive colony show significant overlap [3], but neither of these sets of genes overlapped with those modulated by aggression experienced during pre-adult development. This discrepancy could reflect differences in the stability of social effects experienced at these two different life stages. Socially-induced changes in aggression during adulthood are reversible [2, 64, 79], while effects induced during the pre-adult stages are relatively stable, present 1 week into adulthood, even when bees were kept in a common laboratory environment [66]. Consistent with this hypothesis, the greatest degree of overlap between our gene expression results and previous aggression studies is with the shift in aggression associated with behavioral maturation in adult worker bees. This protracted shift in aggression is the most intransigent of all environmentally induced shifts in behavior evaluated in Alaux et al. [3].

Limited overlap in molecular signatures across aggression studies could reflect the fact that socially-induced changes in behavior result from regulatory mechanisms at more than one level of biological organization. For example, behavioral maturation is associated with large-scale brain structural changes that are less dynamic than brain molecular changes [92]. Similarly, variation in the honey bee developmental environment is known to cause changes in adult brain structure [34]. It is feasible that behavioral variation in our study, like adult behavioral maturation, reflects dynamic processes at multiple interacting levels of biological organization that differ in their relative plasticity [68]. An alternative hypothesis is that variation in aggression associated with the developmental environment is fundamentally different than adult plasticity, because for example, the experience affects only a subset of neuronal populations that regulate aggression [45].

In the current results, changes in brain molecular state are accompanied by shifts in gene expression in both the fat body and midgut. This result is consistent with patterns of sickness behavior in other animals, where molecular signals of peripheral infection impact aggression-relevant signaling in the brain [57]. In the honey bee, no previous study of aggression has assessed molecular variation in peripheral tissues, although recent work suggests there may be some common master regulatory genes associated with age-related behavioral changes across diverse tissues in the honey bee [5, 44]. In our study, brain gene expression changes were modest relative to the fat body and midgut, and perhaps as a result, we found only a single gene that was differentially expressed across all three tissues. Because this gene, GB51409, is a homeobox transcription factor (*Nkx-6.1*), it may indeed serve as a master regulator of molecular state. However, it was not identified as such across in a recent age-related comparison of tissue-specific gene expression in Johnson and Jasper [44]. Particularly comparing the fat body and midgut, genes that were differentially expressed as a function of aggression showed concordance in direction change, consistent with the possibility that a systemic signal is regulating tissue molecular state generally across the organism. Future work will investigate correlated expression across tissue types, the factors that coordinate the infection-like molecular state across tissues, and the relationship between baseline aggression and susceptibility to infection as a result of tissue-specific and tissue-independent processes.

Aggression is easy to rapidly assess at the colony level [66]; future work should consider how it is mechanistically related to other phenotypes that impact colony success. Aggression is an energy-intensive high-performance phenotype sometimes positively correlated with foraging activity at the colony level [69, 94], suggesting foraging effort may shift concurrently with changes in aggression. Foraging behavior is impacted by individual health, but like aggression, it is also modulated by social cues [77], raising the possibility that social responsiveness is altered in low-aggression or diseased individuals. A recent study in honey bees showed that individuals exhibit different levels of social responsiveness, showing high or low levels of response to cues, whether or not these cues matched individual behavioral specialization [78]. Similarly, chronic stress impacts how individuals respond to social cues in the context of aggression [64]. Behavioral variation could reflect individual variation in response thresholds to sensory stimuli. In keeping with this idea, we find that differentially expressed genes as a function of aggression in the current study are enriched for processes related to sensory development. A relationship between sensory response, aggression, and health may explain why high aggression colonies are more effective at removing *Varroa* mites, which are typically detected using olfactory information [66, 73].

Social cohesion is critical to honey bee colony health. The relationship between social behaviors and sickness is complex: social organisms have high levels of conspecific contact, and as a result, many have evolved forms of social immunity, where social interactions are used to prevent or respond to the presence of infectious agents in a social group [38]. Conversely, because social interactions also transmit disease, individuals may avoid or otherwise reject infected individuals [8]. Honey bees exhibit both positive and negative social responses to infected nestmates [19, 63]. Individual infection, on the other hand, impacts foraging behavior and learning and memory [30], but it is unknown if it generally impacts social response or cue sensitivity. Understanding how aggression relates to other social behaviors in the context of infection is an important area of future study.

## Conclusions

Molecular evidence suggests that low aggression honey bees, though otherwise healthy, show a physiological state that resembles infection or stress. In the honey bee, where multiple stressors increase mortality risk by acting in concert on the same physiological pathways within individuals, a physiological phenotype that resembles infection may increase the severity of the health consequences of additional stressors. A diseased bee, when faced with additional insults, is likely to show a cumulative health effect that is more extreme than a healthy bee. Likewise, low aggression bees are more likely to show negative health impacts of disease and other stressors compared to high aggression bees due to their disease-like state. As in vertebrate species, behavior could be used to predict resilience to health stressors in the honey bee. Links between aggression and disease resilience in the honey bee should be considered in the context of future management and breeding efforts aimed at improving health outcomes.

## Methods

### Honey bee tissue samples

Samples for sequencing were a subset of specimens from a previously published study performed during summer 2013 and 2014. In this study we showed that workers introduced into high-aggression hives as 0–24 h old eggs, and kept in these hives through the pupal stage, were more aggressive as adults compared to siblings housed in low-aggression hives. The more aggressive bees also showed increased pesticide tolerance. We demonstrated that behavioral effects were robust across 18 unique colonies (9 high and 9 low aggression) using sibling workers derived from 15 queens (siblings from 14 of 15 queens showed the same trend of developmental effects). This sample reflects three different experiments conducted across 2 years and two geographic locations, Illinois and Pennsylvania, at three times during the summer [66].

The samples used in the current study (preserved from one of the experiments above) were siblings from a single queen kept in one high and one low aggression hive. The two hives had equivalent mite loads (5 mites per colony, measured on a sticky board [66];), were kept in the same apiary, and originated from the same commercial source. Our approach here, in which we perform a molecular assessment for a small subset of individuals from a much larger behavioral dataset, reflects a strategy typical of transcriptomic studies of behavior, especially in social insects [3, 26, 74, 85, 88]. Sub-sampling is employed even in studies of hive-level phenotypic variation because gene expression replication is at the level of the individual bee. This sub-sampling approach resembles a strategy typical of studies assessing individual behavioral variation within a social group [10, 48].

Sub-sampling is particularly relevant in the current molecular analysis, as behavioral and physiological results from our prior study were highly consistent across hives and genotypes [66]. Furthermore, because the queen mother of the siblings sequenced in the current analysis was outbred and naturally-mated (honey bee queens mate with 17–20 males [82];), the results are generalizable to more than one genetic background, as individuals were a mixture of full and half-siblings. It is important to note that one short-coming of our sub-sampling strategy is that we cannot say definitively that the molecular differences we observe are solely a result of the level of aggression displayed by nestmates during development. They could arise due to some other feature of the hive that is not representative of the broader phenotypic effects we observed in our previous study [66]. *Varroa* mite presence is unlikely to be an important difference (see above). Also, our results demonstrate that pathogen infection is an unlikely source of phenotypic variation.

For our two target hives used in the current molecular study, honeycomb frames containing pupating workers were removed from the hives 1 day prior to adult emergence (calculated based on known worker honey bee developmental timing [91];) and allowed to emerge in a laboratory incubator kept at 34 °C. Once workers emerged, some were set aside for molecular analysis (~ 30 individuals) and others were kept in small groups (6 bees per group) for aggression assays. We used different individual bees for the behavioral and molecular assays because the experience of an aggression assay causes extensive and lasting changes in gene expression [3, 65, 79], which, in our case, could obscure the developmental effects we were targeting. All bees were kept in an incubator and fed 50% sucrose until they were 8 days old [64, 69]. This approach allowed us to isolate the behavioral and molecular effects of the developmental environment, since all bees experienced a common laboratory environment for a prolonged time period as an adult. On day 8 of adulthood, the bees in smaller groups were assayed for aggression by measuring aggressive behaviors displayed towards a foreign bee introduced to the group [12]. Groups of siblings raised in high aggression colonies displayed higher aggression per individual bee than groups of siblings kept in low aggression colonies. The bees collected for molecular analysis were then killed in a − 20 °C freezer and transferred to a − 80 °C freezer for long-term storage (please note that it is possible that this method of killing the bees could add variation in gene expression profiles). Thus, the molecular analysis in the current study assesses individuals drawn from a larger group for which we collected behavioral data. The behavioral data reflected the pattern in our larger study, that development in a high aggression hive is correlated with increased aggression once bees reach adulthood.

We dissected brains and midguts by submerging heads and abdominal tissues in chilled RNAlater ICE (Thermo Fisher Scientific Waltham, MA, USA) [26, 65]. Additional tissues (e.g., the sting apparatus) were removed from the abdomen, and fat body RNA was extracted directly from the tissue that remained adhered to the abdominal cuticle. We extracted RNA using the Aurum Fatty and Fibrous RNA kit (Bio-Rad, Hercules, CA, USA, includes on-column DNA digestion). Brains were homogenized using a handheld motorized pestle, while midgut and fat body were homogenized with a bead homogenizer (MP Biomedicals, Santa Ana, CA, USA). RNA was quantified on a plate reader (ClarioStar, BMG Labtech, Ortenberg, Germany) and Bioanalyzer instrument (Agilent Technologies, Santa Clara, CA, USA). Where possible, we retained samples for sequencing for which we had all three tissues from a single individual, and where the RNA Integrity Number was greater than 7. The final sequencing results include *N* = 11 individuals from each colony with all three tissues sequenced, and *N* = 1 low aggression and *N* = 2 high aggression individuals with the brain and midgut only sequenced (72 samples total).

### Sequencing, mapping, and differential expression analysis

Library construction (stranded mRNA TruSeq libraries) and sequencing (Illumina HiSeq 4000, 50 bp reads, 12 samples pooled per lane) was performed by the Duke University Sequencing and Genomic Technologies Shared Resource. We processed reads using Trimmomatic (v. 0.36, default parameters) to remove Illumina sequence adaptors and trim low quality bases. Reads were aligned to the *Apis mellifera* genome (version 4.5, downloaded on August 82,018 from the Ensembl database) using HiSat 2.1.0 [47], and we used HTSeq 0.11.1 [7] to calculate read counts on a per-gene basis. Samples averaged 89.6% alignment success (~ 30 million reads per sample). Reads were also assessed for the presence of common honey bee pathogens (see “Pathogen assessment” below). We used the estimateDisp, glmQLFit, and glmQLFTest functions in EdgeR (v.3.24.3) to evaluate differential expression as a function of hive aggression on a per-tissue basis.

GO terms were assigned to genes with Trinotate v3.0.1 [32] using the standard approach incorporating comparisons with the SwissProt database using BLASTX and BLASTP [4] and the Pfam database [62] using hmmscan [22]. Signal peptides and transmembrane helices were predicted with signalP [59] and TMHMM [49], respectively. Enrichment of GO terms in differentially expressed sets of genes was then calculated using GO-TermFinder [11]. *P*-values from GO analyses were corrected using the Benjamini-Hochberg approach.

### Enrichment analyses

To determine whether the molecular signature associated with variation in aggression in our samples resembled other contexts for phenotypic change, e.g., infection, behavioral maturation, or adult exposure to aggression social cues, we performed a series of enrichment tests that evaluated the statistical overlap between our differentially expressed gene lists and gene lists associated with phenotypes of interest from previous studies [3, 18]. Alaux et al. [3] was a microarray study that included data for the brain only, while Doublet et al. [18] was a meta-analysis of predominantly RNAseq datasets that represent assessments of the brain, midgut, fat body, or combinations of tissues containing one or more of our sampled tissues. We chose to compare our results to Alaux et al. [3] because they evaluated gene expression in several contexts for variation in aggression within a single study. Thus, we could robustly evaluate several hypotheses with our data without technical biases associated with comparing gene sets across distinct aggression studies with variable analytical approaches. To remain consistent with previous studies [3], we filtered our brain gene expression list for genes highly expressed in the hypopharyngeal gland, a possible source of contamination, prior to enrichment tests [65]. For comparisons to Alaux et al. [3], microarray probes were converted to BeeBase ID numbers [65], and for comparison to Doublet et al. [18], BeeBase IDs identified in our current study were converted to RefSeq IDs using NCBI Batch Entrez. Differences in gene identities and methods across studies decreased the size of the gene universe for enrichment analyses, and all analyses accounted for this change. We performed hypergeometric tests for enrichment using the phyper function in R [88]. Tests for significant bias in direction of differential expression were performed using the binom.test function in R.

### Pathogen assessment

We evaluated the relationship between pathogen presence and aggression by estimating the abundance of previously identified honey bee pathogens with our RNAseq data. Reads from each specimen were mapped to a database of known honey bee pathogens with sequenced genomes. This database consisted of the five bacterial pathogens *Melissococcus plutonius* (GCF_000747585.1), *Paenibacillus larvae* (GCF_002003265.1), *Serratia marcescens* (GCF_000513215.1), *Spiroplasma apis* (GCF_000500935.1), and *Spiroplasma melliferum* (GCF_000236085.2)*,* the chalkbrood fungus *Ascosphaera apis (*GCA_000149775.1)*,* the three stonebrood fungi *Aspergillus fumigatus* (GCF_000002655.1)*, A. flavus* (GCF_000006275.2)*,* and *A. niger* (GCF_000002855.3), and the nine honey bee viruses Acute bee paralysis virus (GCF_000856345.1), *Apis mellifera* filamentous virus (GCF_001308775.1), Black queen cell virus (GCF_000851425.1), Chronic bee paralysis virus (GCF_000875145.1), Deformed wing virus (GCF_000852585.1), Israel acute paralysis virus (GCF_000870485.1), Kashmir bee virus (GCF_000853385.1), Sacbrood virus (GCF_000847625.1), and Slow bee paralysis virus (GCF_000887395.1). This list, while not exhaustive, should capture the majority of possible pathogens expected to be present in appreciable frequency [13, 20, 25]. When genomes were represented by multiple scaffolds, we concatenated them into a single sequence for mapping. Reads were mapped to this database using BWA (v.0.7.15) [51] and a single Reads per Kilobase of transcript per million Mapped reads (RPKM) value was calculated for each pathogen genome for each bee specimen. Wilcoxon rank-sum tests were then used to calculate differences in RPKM estimates in each tissue type between high and low aggression hives. Results were corrected for multiple testing (18 total tests) using the Benjamini-Hochberg approach. We also performed χ^2^ tests for each pathogen to determine if their presence, rather than abundance, was associated with aggressive behavior. The pathogen was counted as present if its RPKM value was greater than the 10th percentile of the RPKM’s across all samples for that pathogen. Again, the resulting *p*-values were corrected using Benjamini-Hochberg.

## Supplementary information

**Additional file 1.** This file contains complete lists of differentially expressed genes in the brain (Table S1), fat body (Table S2), and midgut (Table S3), GO term lists in the brain (Table S4) and fat body (Table S5), pathogen data for the brain (Table S6), fat body (Table S7), and midgut (Table S8), and data accession numbers (Table S9).

## Data Availability

The datasets supporting the conclusions of this article are deposited in the NCBI SRA repository. The BioProject Accession Number is PRJNA562696, and the individual sample SRA numbers are listed in Additional file 1: Table S9 of the Supplemental Material.

## Abbreviations

GO: Gene Ontology
RPKM: Reads Per Kilobase of transcript per Million mapped reads

## References

[1] Adamo SA, Jensen M, Younger M (2001). Changes in lifetime immunocompetence in male and female Gryllus texensis (formerly G. integer): trade-offs between immunity and reproduction. Anim Behav.

[2] Alaux C, Robinson GE (2007). Alarm pheromone induces immediate-early gene expression and slow behavioral response in honey bees. J Chem Ecol.

[3] Alaux C, Sinha S, Hasadsri L, Hunt GJ, Guzman-Novoa E, Degrandi-Hoffman G, Uribe-Rubio JL, Southey BR, Rodriguez-Zas S, Robinson GE (2009). Honey bee aggression supports a link between gene regulation and behavioral evolution. Proc National Acad Sci USA.

[4] Altschul SF, Gish W, Miller W, Myers EW, Lipman DJ (1990). Basic local alignment search tool. J Mol Biol.

[5] Ament SA, Wang Y, Chen CC, Blatti CA, Hong F, Liang ZS, Negre N, White KP, Rodriguez-Zas SL, Mizzen CA, Sinha S, Zhong S, Robinson GE (2012). The transcription factor ultraspiracle influences honey bee social behavior and behavior-related gene expression. PLoS Genet.

[6] Ament SA, Wang Y, Robinson GE (2010). Nutritional regulation of division of labor in honey bees: toward a systems biology perspective. Wiley Interdiscip Rev Syst Biol Med.

[7] Anders S, Pyl PT, Huber W (2015). HTSeq--a Python framework to work with high-throughput sequencing data. Bioinformatics.

[8] Arakawa H, Arakawa K, Deak T (2010). Oxytocin and vasopressin in the medial amygdala differentially modulate approach and avoidance behavior toward illness-related social odor. Neuroscience.

[9] Biesmans S, Meert TF, Bouwknecht JA, Acton PD, Davoodi N, De Haes P, Kuijlaars J, Langlois X, Matthews LJ, Ver DL, Hellings N, Nuydens R (2013). Systemic immune activation leads to neuroinflammation and sickness behavior in mice. Mediat Inflamm.

[10] Biesmeijer JC, Seeley TD (2005). The use of waggle dance information by honey bees throughout their foraging careers. Behav Ecol Sociobiol.

[11] Boyle EI, Weng S, Gollub J, Jin H, Botstein D, Cherry JM, Sherlock G (2004). GO::TermFinder--open source software for accessing gene ontology information and finding significantly enriched gene ontology terms associated with a list of genes. Bioinformatics.

[12] Breed MD, Williams KR, Fewell JH (1988). Comb wax mediates the acquisition of nest-mate recognition cues in honey bees. Proc Natl Acad Sci.

[13] Brutscher LM, McMenamin AJ, Flenniken ML (2016). The buzz about honey bee viruses. PLoS Pathog.

[14] Burdick NC, Randel RD, Carroll JA, Welsh TH (2011). Interactions between temperament, stress, and immune function in cattle. Int J Zool.

[15] Camazine SM (1986). Differential reproduction of the mite, *Varroa jacobsoni* (Mesostigmata: Varroidae), on Africanized and European honey bees (Hymenoptera: Apidae). Ann Entomol Soc Am.

[16] Chandrasekaran S, Rittschof CC, Djukovic D, Gu H, Raftery D, Price ND, Robinson GE (2015). Aggression is associated with aerobic glycolysis in the honey bee brain(1). Genes Brain Behav.

[17] Dantzer R (2009). Cytokine, sickness behavior, and depression. Immunol Allergy Clin N Am.

[18] Doublet V, Poeschl Y, Gogol-Doring A, Alaux C, Annoscia D, Aurori C, Barribeau SM, Bedoya-Reina OC, Brown MJ, Bull JC, Flenniken ML, Galbraith DA, Genersch E, Gisder S, Grosse I, Holt HL, Hultmark D, Lattorff HM, Le Conte Y, Manfredini F, McMahon DP, Moritz RF, Nazzi F, Nino EL, Nowick K, van Rij RP, Paxton RJ, Grozinger CM (2017). Unity in defence: honeybee workers exhibit conserved molecular responses to diverse pathogens. BMC Genomics.

[19] Evans JD, Spivak M (2010). Socialized medicine: individual and communal disease barriers in honey bees. J Invertebr Pathol.

[20] Evison SEF, Jensen AB (2016). The biology and prevalence of fungal diseases in managed and wild bees. Current Opinion Insect Sc.

[21] Fine JD, Cox-Foster DL, Mullin CA (2017). An inert pesticide adjuvant synergizes viral pathogenicity and mortality in honey bee larvae. Sci Rep.

[22] Finn RD, Clements J, Eddy SR (2011). HMMER web server: interactive sequence similarity searching. Nucleic Acids Res.

[23] Fogsgaard KK, Rontved CM, Sorensen P, Herskin MS (2012). Sickness behavior in dairy cows during Escherichia coli mastitis. J Dairy Sci.

[24] Fujiyuki T, Takeuchi H, Ono M, Ohka S, Sasaki T, Nomoto A, Kubo T (2004). Novel insect Picorna-like virus identified in the brains of aggressive worker honeybees. J Virol.

[25] Funfhaus A, Ebeling J, Genersch E (2018). Bacterial pathogens of bees. Current Opin Insect Sci.

[26] Galbraith DA, Yang X, Nino EL, Yi S, Grozinger C (2015). Parallel epigenomic and transcriptomic responses to viral infection in honey bees (Apis mellifera). PLoS Pathog.

[27] Gibson JD, Arechavaleta-Velasco ME, Tsuruda JM, Hunt GJ (2015). Biased allele expression and aggression in hybrid honeybees may be influenced by inappropriate nuclear-cytoplasmic signaling. Front Genet.

[28] Giray T, Guzman-Novoa E, Aron CW, Zelinsky B, Fahrbach SE, Robinson GE (2000). Genetic variation in worker temporal polyethism and colony defensiveness in the honey bee *Apis mellifera*. Behav Ecol.

[29] Goblirsch M, Huang ZY, Spivak M (2013). Physiological and behavioral changes in honey bees (Apis mellifera) induced by Nosema ceranae infection. PLoS One.

[30] Gomez-Moracho T, Heeb P, Lihoreau M (2017). Effects of parasites and pathogens on bee cognition. Ecol Entomol.

[31] Goulson D, Nicholls E, Botias C, Rotheray EL (2015). Bee declines driven by combined stress from parasites, pesticides, and lack of flowers. Science.

[32] Grabherr MG, Haas BJ, Yassour M, Levin JZ, Thompson DA, Amit I, Adiconis X, Fan L, Raychowdhury R, Zeng Q, Chen Z, Mauceli E, Hacohen N, Gnirke A, Rhind N, di Palma F, Birren BW, Nusbaum C, Lindblad-Toh K, Friedman N, Regev A (2011). Full-length transcriptome assembly from RNA-Seq data without a reference genome. Nat Biotechnol.

[33] Gregory PG, Evans JD, Rinderer T, de Guzman L (2005). Conditional immune-gene suppression of honeybees parasitized by *Varroa* mites. J Insect Sci.

[34] Groh C, Tautz J, Rossler W (2004). Synaptic organization in the adult honey bee brain is influenced by brood-temperature control during pupal development. Proc National Acad Sci USA.

[35] Guzman-Novoa E, Hunt GJ, Uribe-Rubio JL, Prieto-Merlos D (2004). Genotypic effects of honey bee (Apis mellifera) defensive behavior at the individual and colony levels: the relationship of guarding, pursuing and stinging. Apidologie.

[36] Guzman-Novoa E, Page RE (1994). Genetic dominance and worker interactions affect honeybee colony defense. Behav Ecol.

[37] Harbo JR, Harris JW (1999). Heritability in honey bees (Hymenoptera: Apidae) of characteristics associated with resistance to *Varroa jacobsoni* (Mesostigmata: Varroidae). J Econ Entomol.

[38] Hennessy MB, Deak T, Schiml PA (2014). Sociality and sickness: have cytokines evolved to serve social functions beyond times of pathogen exposure?. Brain Behav Immun.

[39] Hodes GE, Pfau ML, Leboeuf M, Golden SA, Christoffel DJ, Bregman D, Rebusi N, Heshmati M, Aleyasin H, Warren BL, Lebonte B, Horn S, Lapidus KA, Stelzhammer V, Wong EH, Bahn S, Krishnan V, Bolanos-Guzman CA, Murrough JW, Merad M, Russo SJ (2014). Individual differences in the peripheral immune system promote resilience versus susceptibility to social stress. Proc Natl Acad Sci U S A.

[40] Huang Z, Robinson GE (1992). Honeybee colony integration: worker-worker interactions mediate hormonally regulated plasticity in division of labor. Proc Natl Acad Sci.

[41] Huang ZY, Wang Y, The Hive and the Honey bee (2015). Social physiology of honey bees: differentiation in behaviors, castes, and longevity - part 2. By a.M. Graham.

[42] Hunt GJ, Guzman-Novoa E, Fondrk MK, Page RE (1998). Quantitative trait loci for honey bee stinging behavior and body size. Genetics.

[43] Hunt GJ, Guzmán-Novoa E, Uribe-Rubio JL, Prieto-Merlos D (2003). Genotype–environment interactions in honeybee guarding behaviour. Anim Behav.

[44] Johnson BR, Jasper WC (2016). Complex patterns of differential expression in candidate master regulatory genes for social behavior in honey bees. Behav Ecol Sociobiol.

[45] Kabelik D, Klatt JD, Kingsbury MA, Goodson JL (2009). Endogenous vasotocin exerts context-dependent behavioral effects in a semi-naturalistic colony environment. Horm Behav.

[46] Kazlauskas N, Klappenbach M, Depino AM, Locatelli FF (2016). Sickness behavior in honey bees. Front Physiol.

[47] Kim D, Langmead B, Salzberg SL (2015). HISAT: a fast spliced aligner with low memory requirements. Nat Methods.

[48] Klein BA, Olzsowy KM, Klein A, Saunders KM, Seeley TD (2008). Caste-dependent sleep of worker honey bees. J Exp Biol.

[49] Krogh A, Larsson B, von Heijne G, Sonnhammer EL (2001). Predicting transmembrane protein topology with a hidden Markov model: application to complete genomes. J Mol Biol.

[50] Leoncini I, Le Conte Y, Costagliola G, Plettner E, Toth AL, Wang M, Huang Z, Becard JM, Crauser D, Slessor KN, Robinson GE (2004). Regulation of behavioral maturation by a primer pheromone produced by adult worker honey bees. Proc Natl Acad Sci U S A.

[51] Li H, Durbin R (2009). Fast and accurate short read alignment with burrows-Wheeler transform. Bioinformatics.

[52] Li-Byarlay H, Rittschof CC, Massey JH, Pittendrigh BR, Robinson GE (2014). Socially responsive effects of brain oxidative metabolism on aggression. Proc Natl Acad Sci U S A.

[53] Maes M, Berk M, Goehler L, Song C, Anderson G, Galecki P, Leonard B (2012). Depression and sickness behavior are Janus-faced responses to shared inflammatory pathways. BMC Med.

[54] Mao W, Schuler MA, Berenbaum MR (2013). Honey constituents up-regulate detoxification and immunity genes in the western honey bee *Apis mellifera*. Proc National Academy of Sciences USA.

[55] McMenamin A.J. & Genersch E. Honey bee colony losses and associated viruses. Curr Opin Insect Sci. 2015;8:121–9. 10.1016/j.cois.2015.01.015 32846659

[56] Mortensen AN, Ellis JD. The effects of artificial rearing environment on the behavior of adult honey bees, *Apis mellifera* L. Behavioral Ecol Sociobiol. 2018;72:92.

[57] Nelson RJ, Chiavegatto S (2001). Molecular basis of aggression. Trends Neurosci.

[58] Nouvian M, Reinhard J, Giurfa M (2016). The defensive response of the honeybee *Apis mellifera*. J Exp Biol.

[59] Petersen TN, Brunak S, von Heijne G, Nielsen H (2011). SignalP 4.0: discriminating signal peptides from transmembrane regions. Nat Methods.

[60] Preston SR, Palmer JH, Harrison JW, Carr HM, Rittschof CC (2019). The impacts of maternal stress on worker phenotypes in the honey bee. Apidologie.

[61] Proudfoot KL, Weary DM, von Keyserlingk MAG (2012). Linking the social environment to illness in farm animals. Appl Anim Behav Sci.

[62] Punta M, Coggill PC, Eberhardt RY, Mistry J, Tate J, Boursnell C, Pang N, Forslund K, Ceric G, Clements J, Heger A, Holm L, Sonnhammer EL, Eddy SR, Bateman A, Finn RD (2012). The Pfam protein families database. Nucleic Acids Res.

[63] Richard FJ, Aubert A, Grozinger CM (2008). Modulation of social interactions by immune stimulation in honey bee, Apis mellifera, workers. BMC Biol.

[64] Rittschof CC (2017). Sequential social experiences interact to modulate aggression but not brain gene expression in the honey bee (*Apis mellifera*). Front Zool.

[65] Rittschof CC, Bukhari SA, Sloofman LG, Troy JM, Caetano-Anolles D, Cash-Ahmed A, Kent M, Lu X, Sanogo YO, Weisner PA, Zhang H, Bell AM, Ma J, Sinha S, Robinson GE, Stubbs L (2014). Neuromolecular responses to social challenge: common mechanisms across mouse, stickleback fish, and honey bee. Proc Natl Acad Sci U S A.

[66] Rittschof CC, Coombs CB, Frazier M, Grozinger CM, Robinson GE (2015). Early-life experience affects honey bee aggression and resilience to immune challenge. Sci Rep.

[67] Rittschof CC, Grozinger CM, Robinson GE (2015). The energetic basis of behavior: bridging behavioral ecology and neuroscience. Curr Opin Behav Sci.

[68] Rittschof CC, Hughes KA (2018). Advancing behavioural genomics by considering timescale. Nat Commun.

[69] Rittschof CC, Robinson GE (2013). Manipulation of colony environment modulates honey bee aggression and brain gene expression. Genes Brain Behav.

[70] Rittschof C.C., Vekaria H.J., Palmer J.H. & Sullivan P.G. Brain mitochondrial bioenergetics change with rapid and prolonged shifts in aggression in the honey bee, Apis mellifera. J Exp Biol. 2018;221:1–10. 10.1242/jeb.176917 29496782

[71] Rittschof CC, Vekaria HJ, Palmer JH, Sullivan PG (2019). Biogenic amines and activity levels alter the neural energetic response to aggressive social cues in the honey bee *Apis mellifera*. J Neurosci Res.

[72] Rortais A, Tentcheva D, Papachristoforou A, Gauthier L, Arnold G, Colin ME, Bergoin M (2006). Deformed wing virus is not related to honey bees' aggressiveness. Virol J.

[73] Rosenkranz P, Aumeier P, Ziegelmann B (2010). Biology and control of Varroa destructor. J Invertebr Pathol.

[74] Schmehl DR, Teal PE, Frazier JL, Grozinger CM (2014). Genomic analysis of the interaction between pesticide exposure and nutrition in honey bees (Apis mellifera). J Insect Physiol.

[75] Schulz DJ, Huang ZY, Robinson GE (1999). Effects of colony food shortage on behavioral development in honey bees. Behav Ecol Sociobiol.

[76] Scofield HN, Mattila HR (2015). Honey bee workers that are pollen stressed as larvae become poor foragers and waggle dancers as adults. PLoS One.

[77] Seeley TD (1997). Honey bee colonies are group-level adaptive units. Am Nat.

[78] Shpigler HY, Saul MC, Corona F, Block L, Cash AA, Zhao SD, Robinson GE (2017). Deep evolutionary conservation of autism-related genes. Proc Natl Acad Sci U S A.

[79] Shpigler HY, Saul MC, Murdoch EE, Cash-Ahmed AC, Seward CH, Sloofman L, Chandrasekaran S, Sinha S, Stubbs LJ, Robinson GE (2017). Behavioral, transcriptomic and epigenetic responses to social challenge in honey bees. Genes Brain Behav.

[80] Smith KM, Loh EH, Rostal MK, Zambrana-Torrelio CM, Mendiola L, Daszak P (2013). Pathogens, pests, and economics: drivers of honey bee colony declines and losses. Ecohealth.

[81] Supek F, Bosnjak M, Skunca N, Smuc T (2011). REVIGO summarizes and visualizes long lists of gene ontology terms. PLoS One.

[82] Tarpy DR, Delaney DA, Seeley TD (2015). Mating frequencies of honey bee queens (Apis mellifera L.) in a population of feral colonies in the northeastern United States. PLoS One.

[83] Toth AL, Kantarovich S, Meisel AF, Robinson GE (2005). Nutritional status influences socially regulated foraging ontogeny in honey bees. J Exp Biol.

[84] Toth AL, Robinson GE (2005). Worker nutrition and division of labour in honeybees. Anim Behav.

[85] Toth AL, Tooker JF, Radhakrishnan S, Minard R, Henshaw MT, Grozinger CM (2014). Shared genes related to aggression, rather than chemical communication, are associated with reproductive dominance in paper wasps (*Polistes metricus*). BMC Genomics.

[86] Wang Y, Campbell JB, Kaftanoglu O, Page RE, Amdam GV, Harrison JF (2016). Larval starvation improves metabolic response to adult starvation in honey bees (*Apis mellifera* L.). J Exp Biol.

[87] Weary DM, Huzzey JM, von Keyserlingk MA (2009). Board-invited review: using behavior to predict and identify ill health in animals. J Anim Sci.

[88] Wheeler MM, Robinson GE (2014). Diet-dependent gene expression in honey bees: honey vs sucrose or high fructose corn syrup. Sci Rep.

[89] Whitfield CW, Band MR, Bonaldo MF, Kumar CG, Liu L, Pardinas JR, Robertson HM, Soares MB, Robinson GE (2002). Annotated expressed sequence tags and cDNA microarrays for studies of brain and behavior in the honey bee. Genome Res.

[90] Wilson-Rich N, Spivak M, Fefferman NH, Starks PT (2009). Genetic, individual, and group facilitation of disease resistance in insect societies. Annu Rev Entomol.

[91] Winston ML (1987). The biology of the honey bee.

[92] Withers GS, Fahrbach SE, Robinson GE (1993). Selective neuroanatomical plasticity and division of labour in the honeybee. Nature.

[93] Woyciechowski M, Moroń D (2009). Life expectancy and onset of foraging in the honeybee (*Apis mellifera*). Insect Soc.

[94] Wray MK, Mattila HR, Seeley TD (2011). Collective personalities in honeybee colonies are linked to colony fitness. Anim Behav.

